# Supplementary material for: Study on awareness and management based health action using video intervention (SAMBHAV) for postpartum depression among mothers attending immunisation clinic in a tertiary medical college hospital: Study protocol
Source: PLoS One. 2024 Apr 3;19(4):e0301357. doi: 10.1371/journal.pone.0301357 (PMC10990171; doi:10.1371/journal.pone.0301357)
Supplement: Attachment — Submitted filename: Reponse to Reviewers_SAMBHAV.docx [file pone.0301357.s002.docx]

**Response to Reviewer’s**

**Reviewer 1:**

| ***Sl No*** | ***Reviewer Comments*** | ***Author’s Response*** | ***Page No*** |
| --- | --- | --- | --- |
| 1. | The manuscript covers an important research topic. An interesting recent published article may be of interested in utilized for comparative analysis:  Chaudhary K, Nepal J, Shrestha K, Karmacharya M, Khadka D, Shrestha A, Shakya PR, Rawal S, Shrestha A. Effect of a social media-based health education program on postnatal care (PNC) knowledge among pregnant women using smartphones in Dhulikhel hospital: A randomized controlled trial. Plos one. 2023 Jan 20;18(1): e0280622 | Thank you for your comment.  We have included the study in the manuscript in the introduction section. | 4 |
| 2. | Discussion: Please kindly highlight more clearly the limitations of the study with a few justifications particularly the generalizability of the findings. | Thank you for your comment.  We have made the changes accordingly | 10 |

**Reviewer 2:**

| ***Sl No*** | ***Reviewer Comments*** | ***Author’s Response*** | ***Pg No*** |
| --- | --- | --- | --- |
| 1. | This is an important study with high practical implication. This proposal seemed to be well structured and well planned | Thank you | No changes |

**Reviewer 3:**

| ***Sl No*** | ***Reviewer Comments*** | ***Author’s Response*** | ***Pg No*** |
| --- | --- | --- | --- |
| This protocol addresses a significant public health concern: poor perinatal mental health exacts a severe and substantial toll borne by women, their families, and the healthcare system. The authors’ intention to address this issue by raising awareness and lessening the stigma of postpartum depression is laudable | | | |
| 1. | Pg. 2 “the percentage of women reporting difficulty in mother child bonding and harm to the baby varied between 5- 77.1% [9,10]” – Suggest revision for clarity; this could be easily misinterpreted that up to 77% of women harm their baby. | Thank you for your comment.  Yes, we agree that the statement has tendency for misinterpretation. Hence, we have modified the sentence. | 2-3 |
| 2. | Pg. 3 – regarding the birth of a female baby as a risk factor for PPD, please clarify whether this is globally or within a particular geographic/cultural context. | Thank you for your comment.  There has been literature from India, China and Egypt indicating female gender of the child as a risk factor for PPD. | 3 |
| 3. | “Postpartum Depression affects the bonding between the mother and her child and also impairs growth and development of the baby. This might predispose her to chronic or recurring depression.” Citation is needed here. Please clarify the link between diminished bonding and its impacts on infant developmental trajectories and how these factors predispose mothers to chronic or recurring depression. Perhaps the phrase “This might predispose her to chronic or recurring depression” is intended to appear after the discussion on barriers to treatment. | Thank you for your comment.  The statement has been revised as per the literature and details of infant development related issues has been added. The citation has been included. | 3 |
| 4. | Pg. 5 While the sample size for the quasi-experimental component of the study (pre and post-testing educational video effectiveness) is justified via power calculations, no rationale is provided for the sample size of the focus groups or expert evaluation of the video scripts. | Thank you for your comment.  The rationale for sample size for focus group discussion and details of evaluation of video script has been added. | 7-8 |
| 5. | Please describe the psychometric properties of both the Postpartum Depression Literacy Scale and the Patient Health Questionnaire 9. Please include any details pertaining to whether these scales have been validated for use within Indian populations (e.g., are they culturally relevant to your target population). In describing the literature search strategies for developing the script for the 2 videos that form the basis of this intervention, the authors should expand on their stated search parameters. For instance, will the search include grey literature as well as peer-reviewed; any limits with respect to publication date? If the authors are looking for literature that encompasses the Indian context, why is the search limited to English-language only? What search strategies will the authors use to access “blogs”? Furthermore, the authors should consider whether the quality of the included articles will be assessed/appraised in some way. | Thank you for your comment.  We have included the details regarding the psychometric properties of both the tools and its use in Indian population.  The details regarding the literature search are also revised as suggested. | 6-7 |
| 6. | Pg. 7 The following passage appears twice on this page: “Focus group discussion will be conducted among the mother’s attending immunization clinics and healthcare professionals including both doctors and non-doctors including Psychiatric Social Work, Clinical Psychology, Nursing, Obstetrics, Paediatrics and health workers. This will help in-depth understanding about postpartum depression at the grass root level. This will also help in finalising the content for the video script which is a crucial step in the study.” – It is unclear whether this refers to two distinct study phases in the study or is repeated in error. | Thank you for the comment.  We have elaborated the process of conducting the FGD. | 7 |
| 7. | In the focus group discussions that will support developing the video script, the authors should provide details about the number participants in each focus group, the length of time of the focus, and how many focus group meetings they anticipate conducting. What participants will comprise each focus groups – e.g., mothers and health professionals interviewed separately or together? What is the nature or the questions that will guide the focus group discussions & what setting will these meetings occur? | Thank you for the comment.  We have elaborated the process of conducting the FGD. | 7 |
| 8. | Pg. 8: Details regarding the evaluation of the scripts are limited. How will face validity be determined? What is the nature of the data being collected (surveys, qualitative interviews)? How will this data then be analysed? The authors note that experts, mothers, & caregivers will be evaluating the scripts but do not provide details about how many informants will participate. This is also the first mention of “caregivers attending perinatal clinics.” If caregivers are to be included in the study population, then details should be provided in the appropriate section. | Thank you for the comment.  There was a mention about validation of the script and that was purely an oversight.  The script will not be validated and it will be finalized by the internal team of experts. The same has been corrected in the manuscript. | 7-8 |
| 9. | What is the rationale for the 2-month follow-up in administering the postpartum depression literacy scale? Is it expected that mothers will retain the educational information about PPD provided in the videos for that length of time after only one viewing of the video? | Thank you for the comment.  The post test will be conducted at a time interval of 4- 8 weeks after pre-test. The same has been revised in the manuscript. One of the rationales for considering the time interval to be 4-8 weeks between pre-test and post-test  is to look for knowledge retention as immediate posttest is not useful for change in literacy and also to ensure the compliance of contacting the mothers for posttest. As the mothers will be visiting immunization clinics at 6, 10 and 14 weeks for vaccination of the child. The details of same has been included in the manuscript. | 8 |
| 10. | Furthermore, as the other outcome of this video series is for early identification and management of PPD, it also isn’t clear how this will be accomplished after one viewing | Thank you for your comment.  Through this video we aim to educate mothers on various aspects of PPD including subjective experiences and risk factors, stigmatizing factors, barriers and facilitators to access mental health services, this would aid early identification and management of PPD. As a part of the study we will be showing the video only once to the mothers and evaluating the change in literacy status. | 9 |
| 11. | Pg. 9: under statistical analysis “Based on the literature search, important components will be identified to create the script for the video.” – it is unclear what this means. Also, there is no mention of how the PHQ – 9 will be analysed. | Thank you for the comment.  The analysis of findings from the literature search have been revised. The PHQ 9 is used for screening the mothers for PPD and to estimate of prevalence of PPD before and after intervention. The same has been included in the manuscript. | 7, 9 |
| 12. | For the focus group discussion data, the type of thematic analysis is not specified (e.g., Braun & Clark) and the details of the coding strategy are not discussed | Thank you for your comment.  FGDs will be qualitatively analysed. Inductive and thematic theme identification will be done using a descriptive approach. It analysed manually and the themes will be generated based on the codes from the discussion.  The details of the same have been included in the manuscript. | 9 |
| 13. | There do not appear to be any details about recruitment strategies or study setting beyond “immunization clinics. | Thank you for your comment.  Postpartum mothers within 6 months of childbirth attending immunization clinics in Urban and Rural Primary Health Centers. The immunization clinics serve as ideal setting for undertaking any intervention among post-partum mothers, as they visit these clinics at 6, 10, 14 weeks for universal immunization program. Hence this place is ideal for any study planning for intervention. The same has been included in the manuscript. | 5 |

Reviewer 4:

| ***Sl No*** | ***Reviewer’s Comment*** | ***Author’s Response*** | ***Pg No*** |
| --- | --- | --- | --- |
| Thanks for allowing me to review this excellent study protocol. It's quite thorough, but I do advise a few minor modifications: | | | |
| 1. | PLOS ONE says they require a plan for data to be available to readers. You mention your plan for data in the abstract portion as "N/A" since "no results are reported." I do see that in the body of the manuscript (on p1 just before the abstract), you do address data availability differently: "After the study is completed, all relevant data will be made available." Should reconcile these two. | Thank you for your comment.  The sentence has been changed accordingly. | 1 |
| 2. | In the abstract Discussion section line 2, authors mention that the video "can be used to help mothers identify and treat PPD early on improving both maternal and child health outcomes." I appreciate that the video will address knowledge and hence identification of PPD (for mothers and for healthcare staff/workers), but it would not offer treatment. Should therefore remove "and treat" from above. Furthermore, as authors are not measuring health outcomes in this project, I advise a less bold claim in that same sentence regarding outcomes, something like: "...it can be used to help identify mothers' PPD, which can then lead to treatment and improved health outcomes for both mothers and their children." | Thank you for your comment.  If the video intervention is successful in raising awareness of PPD among postpartum mothers and reducing stigma, it can be used to aid early identification of mothers with PPD which can result in early management and improved health outcome for both mothers and children.  The same has been revised in the manuscript. | 2 |
| 3. | Methods and Analysis section, under Exclusion criteria (p5), I presume you will allow mothers to opt out from completing the surveys (PHQ9 and PoDLiS) and from watching the video? You could also add that speaking/reading languages other than English and Kannada are exclusions. | Thank you for your comment.  As we have already considered language of the mothers in the inclusion criteria. We have mentioned the mothers with any of the major illness or mental health issue as one of the exclusion criteria. The same has been changed in the manuscript. | 5 |
| 4. | Methods and analysis section, sample Size (p5): It appears that you are looking to recruit 130 mothers over a period of 27 months? That's just about 5 patients a month. Help me understand (as the reader) why it should be so hard to recruit these mothers? Is it the shortage of postpartum mothers within 6 months of birth attending the immunization clinic? Is it the lack of mothers speaking or reading English or Kannada? Do you anticipate that most mothers will opt out due to the stigma associated with PPD? | Thank you for your comment.  The total study duration is 27 months. The initial 9 months is mainly dedicated for developing the video intervention finalizing the video script and also validation of the tool. The data collection will be undertaken in the next 6 months. And the last 9 months will be used for analysis of the data and report submission. The details of the same is mentioned under the status and timeline section of the manuscript. | 10 |
| 5. | There are several typos I noted, including "et al." which is used multiple times through the research manuscript and should have a period following it. Another example is "immunization" (eg., top of p11) vs. "immunisation." One is a more Indian/British spelling. I believe either is OK, but there should be consistency across the manuscript. Have some colleagues help proofread for such errors slipping through? | Thank you for your comment.  The same has been revised in the manuscript. | 1-3, 10 |
| 6. | Development of Script (pp 7-8): There is no mention of consideration of length of the script and resultant video. Presumably the length would impact the compliance of women with watching it. Consider some mention of balancing the important content with the resultant length | Thank you for your comment.  The video script will be of 10 - 12 pages, with resultant video will be of 12 -15 minutes.  The same has been mentioned in the manuscript. | 7- 8 |
| 7. | Administering the questionnaire to mothers (p8): Please elaborate whether the 2-month delayed post-test is designed to assess retention of the information regarding PPD or whether it is intended to pick up new development of PPD 2-months later? | Thank you for the comment.  The post test will be conducted at a time interval of 4- 8 weeks after pre-test. The same has been revised in the manuscript. One of the rationales for considering the time interval to be 4-8 weeks between pre-test and post-test is to look for knowledge retention as immediate posttest is not useful for change in literacy and to ensure the compliance of contacting the mothers for posttest. As the mothers will be visiting immunization clinics at 6, 10 and 14 weeks for vaccination of the child. As suggested, it also intends to look for any new cases of PPD identified after the intervention. The details of same has been included in the manuscript. | 8 |
| 8. | Consistency across the paper: Sometimes the video intervention is mentioned as a single video (eg., Study Outcome section on pp 8-9), and sometimes as two videos (eg., top of p7 in the Data Collection section). | Thank you for your comment.  A single video of 12- 15 minutes’ duration will be developed. The same has been uniformly mentioned in the manuscript. | 7- 8 |
| 9. | Unclear acronym: "FGDs" in Quantitative Analysis section p9. Maybe I missed it, but I'm unclear what this acronym refers to. Perhaps the focus group discussions (per google search, but I couldn't guess without internet help)? | Thank you for your comment.  Here FGDs refer to focus group discussions. The same has been expanded in the manuscript. | 7 |
| 10. | Limitations section on p10: I believe this should be written in paragraph format, consistent with the rest of the research proposal manuscript. I agree that the study is limited by choosing not to measure any health outcomes. Consider adding one or more survey questions to the 2-month follow up... If a mother does score highly on the PHQ 9 indicating depression, does she intend to seek mental health support services? If so, from where? If not, why? | Thank you for your comment.  The limitation of the study has been revised.  In case a mother scores high on PHQ 9, indicating depression them they will be seen by the trained medical officer (As a part of District Mental Health Program (DMHP), all the PHCs runs ‘Manochaitanya Clinic’ on Tuesdays.) and later mothers will be referred to manochiatanya clinic for further evaluation and treatment by mental health professionals. | 10-11 |
